# An unusual case of primary osteosarcoma of the rib in an adult

**DOI:** 10.4103/0971-5851.68847

**Published:** 2010

**Authors:** Suravi Mohanty, Y. K. Inchara, Julian A. Crasta, Anuradha Ananthamurthy

**Affiliations:** *Department of Pathology, St. John’s Medical College and Hospital, Bangalore ‐ 560 034, India*

**Keywords:** *Adult*, *chest wall tumors*, *flat bones*, *microscopy*, *osteosarcoma*, *ribs*

## Abstract

Primary osteosarcomas are one of the most common malignant bone tumors principally affecting the long bones in children and adolescents. An unusual case of a primary osteoblastic osteosarcoma of the rib in a 42-year-old male is presented here. The patient underwent a wide excision of the tumor and chest wall reconstruction. Although clinically unsuspected in this unusual site, the classic microscopic feature of a ramifying osteoid matrix amidst the tumor cells was diagnostic of an osteosarcoma.

## INTRODUCTION

Osteosarcomas (OS) are the most common primary malignant bone tumors exclusive of multiple myeloma. They principally arise in the metaphyses of the long bones, particularly at the lower end of the femur and upper ends of the tibia and humerus.[1] Short bones, spine, and flat bones, such as, the ribs, scapula, pelvis, and craniofacial bones are less frequently involved. Secondary osteosarcomas may develop in unusual bones, such as, vertebra and flat bones in patients treated with chemotherapy.[1] Primary osteosarcoma of the rib, moreso, occurring in an adult, is extremely rare. We present one such case that posed a diagnostic difficulty, owing to its unusual location.

## CASE REPORT

**Figure 1:**
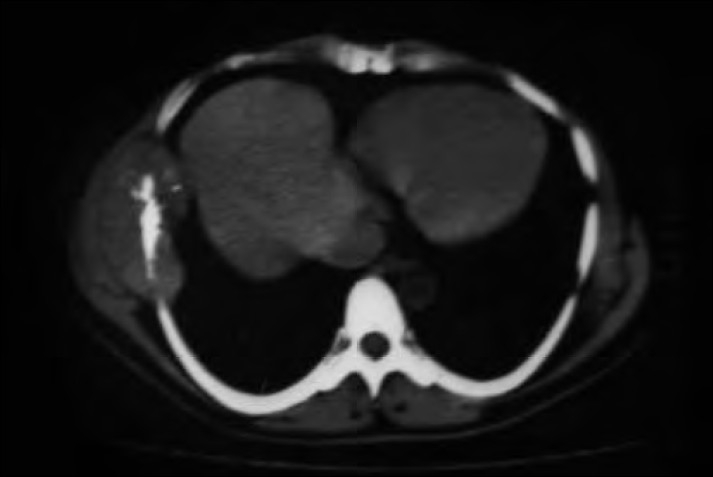
Axial CT image showing a lobulated, soft tissue density mass with calcification and sclerosis, and is seen destroying the rib

A 42-year-old man presented with a progressively increasing right-sided chest wall swelling for three years. The swelling was accompanied by pain for the past three months. However, there was no fever, cough, breathlessness or loss of weight. There was no history of receiving radiation or a pre-existing bone lesion. Physical examination revealed a bony hard mass in the right hemithorax measuring 5×5 cm extending from the anterior axillary to the mid axillary line. An X-ray examination revealed an opacity in the right lateral chest wall involving the ribs and soft tissue. A computed tomography (CT) scan showed a lobulated, extra pleural, soft tissue density, with calcification involving the fifth and sixth ribs, which showed sclerosis and destruction [Figure 1]. The lungs and mediastinum appeared normal. A preoperative diagnosis of chondrosarcoma was considered. A right posterolateral thoracotomy with tumor resection and chest wall reconstruction with prolene mesh was performed. The tumor was excised in toto along with the fourth to eighth ribs.

**Figure 2:**
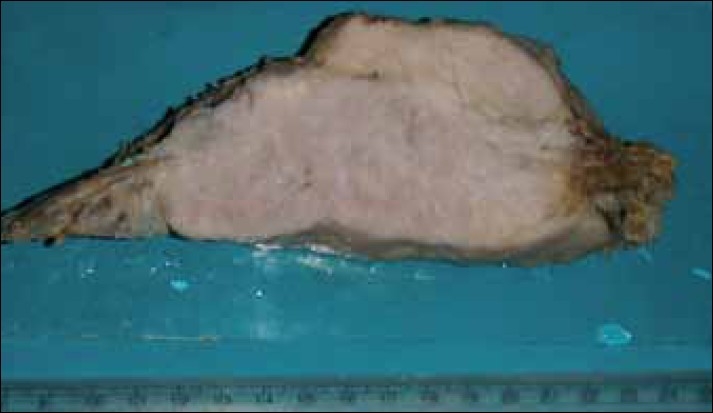
Cut surface of the tumor with attached rib

**Figure 3:**
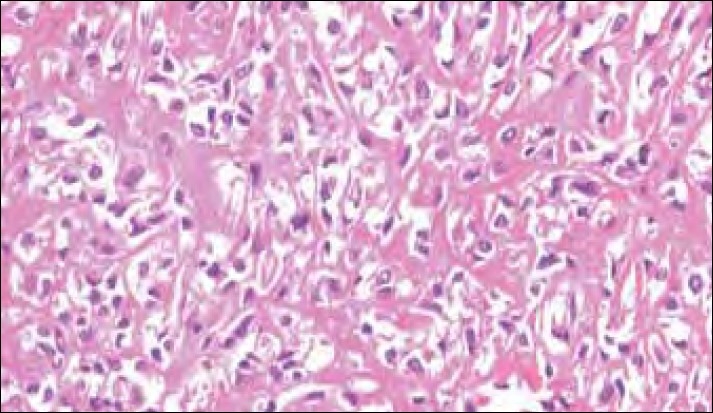
Neoplastic cells rimming haphazardly laid osteoid (H and E, ×200)

Gross examination revealed a partly encapsulated bony hard mass measuring 12×11×4 cm, bearing five ribs. The tumor was seen involving and encasing all the ribs [Figure 2]. The cut surface of the tumor was solid, gray-white, and fleshy. Microscopic examination showed a malignant neoplasm composed of pleomorphic, round-to-spindle shaped cells, which were rimming haphazardly - laid tumor osteoid [Figure 3]. Atypical mitotic figures were also seen. A diagnosis of conventional high grade osteoblastic osteosacroma of the fourth to eighth ribs was made. Although the soft tissue surgical margins were involved, the bone surgical margins were free. The patient was referred to a specialized cancer center for further adjuvant therapy.

## DISCUSSION

Primary osteosarcomas usually originate in the metaphysis of the long bones. Approximately 10% of osteosarcomas are located in the flat bones, with the pelvis being the main site, and a mere 1 – 2% occur in the thoracic bones inclusive of the ribs, sternum, and clavicle.[1] OS are known to have a predilection to affect long bones, such as, the distal femur and proximal tibia, because these are the sites of greatest bone growth, where bone cell mitotic activity is at its peak. In fact there is a high incidence of these tumors in large dog breeds such as Great Danes and St. Bernards, for the same reason.[2]

Cases originating from the ribs are infrequent and have been reported mainly in the pediatric population[3–5] In a large study comprising of 49 cases of primary malignant chest wall tumors there were no osteosarcomas reported.[6] Involvement of flat bones may be seen as a metastatic process or secondary to chemotherapy, but a primary OS is rare.[7]

OS originating from such a rare site poses a diagnostic challenge to the radiologist, pathologist, and the surgeons. The typical ‘sunburst’ radiological pattern observed in the OS of the long bones may not be evident in the OS of the flat bones.[6] Owing to the exclusive site and varying radiological images, these tumors can be confused with other bone lesions and the differentials may include chondrosarcoma, fibrosarcoma or metastatic tumor.

Although a CT scan and magnetic resonance imaging (MRI) can evaluate the exact location and extent of involvement of the bone and adjacent structures, they may not be useful in defining the exact nature of the tumor. Some authors opine that osteosarcoma should be suspected if the CT scan reveals a dense calcification within a mass that is centered in a rib.[8] Histopathological diagnosis is imperative in instituting a definite therapy. The classic feature of a ramifying osteoid matrix laid down by the neoplastic cells clinches the diagnosis and enables one to exclude all the other possible differentials. Diagnostic difficulties may be encountered when the osteoid production is scant, when a diligent search for the same and extensive sampling of the tumor is warranted. A combined effort with radiological and clinical correlation is trustworthy and may serve to avoid pitfalls in the diagnosis. Conventional osteosarcomas are the most aggressive osseous neoplasms. The overall prognosis of osteosarcoma in flat bones remains poor because of the difficulty of complete excision. The guidelines for management and the prognosis and survival rates in rib primary OS is not clear due to the small number of cases studied. However, there is documentation of better survival in patients who have had a complete resection of their tumor at the time of surgery.[4] A local wide excision with removal of the involved ribs and subsequent reconstruction using a mesh followed by adjuvant chemotherapy and radiotherapy may improve survival in these patients.[4] Although OS of flat bones is rarely associated with metastasis, it is prudent to include a CT scan of the thorax, chest x-rays, and bone scans as part of the management protocol, in order to look for metastasis.[4]

In conclusion, this case highlights the fact that OS of the rib, although rare, should be considered in the differential diagnosis of primary malignant neoplasms of the rib.
